# Simple In Vitro ^18^O Labeling for Improved Mass Spectrometry-Based Drug Metabolites Identification: Deep Drug Metabolism Study

**DOI:** 10.3390/ijms24054569

**Published:** 2023-02-26

**Authors:** Boris Tupertsev, Sergey Osipenko, Albert Kireev, Eugene Nikolaev, Yury Kostyukevich

**Affiliations:** 1 Center of Molecular and Cellular Biology (CMCB), Skolkovo Institute of Science and Technology, Nobel Str., 3, 121205 Moscow, Russia; 2 Moscow Institute of Physics and Technology, Phystech School of Biological and Medical Physics, Institutskiy per., 9, Dolgoprudny, 141701 Moscow, Russia; 3 Center for Computational and Data-Intensive Science and Engineering, Skolkovo Institute of Science and Technology, Nobel Str., 3, 121205 Moscow, Russia

**Keywords:** mass spectrometry, drug metabolism, ^18^O, metabolites identification

## Abstract

The identification of drug metabolites formed with different in vitro systems by HPLC-MS is a standard step in preclinical research. In vitro systems allow modeling of real metabolic pathways of a drug candidate. Despite the emergence of various software and databases, identification of compounds is still a complex task. Measurement of the accurate mass, correlation of chromatographic retention times and fragmentation spectra are often insufficient for identification of compounds especially in the absence of reference materials. Metabolites can “slip under the nose”, since it is often not possible to reliably confirm that a signal belongs to a metabolite and not to other compounds in complex systems. Isotope labeling has proved to be a tool that aids in small molecule identification. The introduction of heavy isotopes is done with isotope exchange reactions or with complicated synthetic schemes. Here, we present an approach based on the biocatalytic insertion of oxygen-18 isotope under the action of liver microsomes enzymes in the presence of ^18^O_2_. Using the local anesthetic bupivacaine as an example, more than 20 previously unknown metabolites were reliably discovered and annotated in the absence of the reference materials. In combination with high-resolution mass spectrometry and modern methods of mass spectrometric metabolism data processing, we demonstrated the ability of the proposed approach to increase the degree of confidence in interpretating metabolism data.

## 1. Introduction

The path from a molecule in a laboratory test tube to a drug is time-consuming and expensive [1]. According to the US Congressional Budget Office for 2021 [2], pharmaceutical companies spent $89 billion on R&D in 2019, and this is justified: in addition to the stages of searching for a target and selecting a pool of potentially active molecules, it is necessary to not only empirically confirm the action but also prove the safety of the future drug. The study of metabolism is an integral part of several stages of the research of a drug candidate, the foundation of the evidence base for its safety.

Drug metabolism is a complex biotransformation process whereby drugs are structurally modified by various metabolizing enzymes. Studies on drug metabolism are a key component of processes to, for example, optimize lead compounds for optimal PK/PD properties, identify new chemical entities based on the finding of active metabolites, minimize potential safety liabilities due to the formation of reactive or toxic metabolites, and compare preclinical metabolism in animals with humans for ensuring potential adequate coverage of human metabolites in animals and for supporting human dose prediction [3]. In vitro metabolism studies in human and animal tissue (e.g., liver) preparations and/or in vivo metabolism studies in animals are useful approaches for identifying major metabolism pathways (“soft spots”) of drugs [4]. 

Hepatic metabolism is the primary elimination mechanism for the majority of drugs as well as other xenobiotics and endogenous compounds [5]. One of the most popular in vitro systems for modeling hepatic metabolic pathways of a drug candidate is liver microsomes (LM). Microsomes are closed vesicles in the membrane of the endoplasmic reticulum in liver cells (from mice, rats and humans) that contain a number of phases I and II metabolic enzymes, including the monooxygenase enzymatic complex that comprises cytochrome P450 and its NADPH-dependent reductase [6]. Wide application of LM is caused by their reproducible nature, long-term storage capacity, extensive characterization of optimal incubation conditions and relatively low price [7]. 

Sample analysis after incubation of a substance with microsomes is most often carried out with high-performance liquid chromatography combined with high-resolution mass spectrometry (HPLC-HRMS). This combination is used not only because of its very low limit of detection, good selectivity, accuracy and precision of quantification but also due to accurate mass measurement, which simplifies the interpreting of the results of metabolic studies [8]. Further data processing is carried out considering the structure of the drug and the main metabolic pathways: oxidation, reduction, hydrolysis, cyclization/decyclization for phase I modifications and conjugations such as acetylation, sulphation and glucuronidation for phase II metabolism [9]. This stage can be automated using various software, both commercial, such as, Compound Discoverer^TM^ (Thermo Fisher Scientific, Houston, TX, USA) [10], and free, such as MIDAS [11], MetFrag [12] and PyFragMS [13].

Despite the emergence of various software and databases, the identification of compounds is still a complex task. Measurement of the accurate mass, correlation of chromatographic retention times and fragmentation spectra are often insufficient for identification of compounds [14]. Metabolite signals with low intensity “slip under the nose”, since it is often not possible to reliably confirm that a signal belongs to a metabolite and not to other compounds in complex systems. For reliable identification, it is necessary to increase the concentration of the analyte and carry out countersynthesis. Carrying out parallel experiments with and without inserting isotope labels proved to be a tool for aiding small molecule identification [14].

Stable isotopes have already been used in mass spectrometry for better identification of compounds; the main ones are ^1^H/D and ^16^O/^18^O exchanges. Hydrogen/deuterium exchange mass spectrometry (HDX-MS) is widely used in studying proteins [15], peptides [16] and small molecules [14,17]. During HDX, hydrogens on heteroatoms (O, N, S) are replaced by deuterium, which results in an increase of molecular mass according to a number of exchangeable hydrogen atoms present in a given molecule. Via ^1^H/D exchange D. Liu et al. managed to distinguish the S-oxidation metabolite structures from hydroxylation due to two exchanges instead of three [18]. The structures of the carbamoyl glucuronide and N-glucuronide metabolites were also confirmed by this method during biotransformation studies performed on an investigational compound. In the work of E. Padilha on the study of the optimal structure of drugs for the treatment of fibrodysplasia ossificans, progressiva HDX-MS was used for identification structures of drug candidate metabolites [19]. With this approach, it was possible to distinguish N-oxide/hydroxylamine metabolites with one ^1^H/D exchange and hydroxy-metabolites with two ^1^H/D exchanges. Despite the efficiency of the HDX-MS method, its applicability is limited by several factors, the main of which is the high rate of exchange reactions (both direct and reverse) [20]. Thus, isotopically labeled standards cannot be stored. The high reaction rate allows and at the same time makes necessary carrying out online ^1^H/D exchange, which requires refinement of HPLC-MS systems and relatively high consumption of deuterium oxide. A 2003 Pfizer patent for online exchange proposed additionally installing an LC-pump and supplying D_2_O in a ratio of 1:1 to 3:5 eluent and deuterium oxide, respectively [21].

^16^O/^18^O–exchange mass spectrometry is used in proteomics as well as for identification of small molecules. X. Ye et al. described the application of enzyme-catalyzed oxygen exchange in the presence of H_2_^18^O for the qualitative and quantitative determination of proteins [22]. During the reaction, two ^16^O atoms of the C-terminal carboxyl group of proteolytic fragments are typically replaced by two ^18^O atoms, and a stable analyte is obtained. Recently, interest in the use of oxygen exchange for the mass spectrometric study of small molecules has increased. L. Rumiantseva et al. showed the applicability of ^16^O/^18^O exchange for the mass spectrometry determination of carbohydrates [23]. In the work of Osipenko et al., ^16^O/^18^O exchange was performed in urine spiked with drugs, and the results showed that oxygen exchange provides 62% search space reduction for a panel of drug molecules [24]. Oxygens of the carbonyl and carboxylic groups, hydroxyl groups in the allyl position, hydroxyl groups of carbohydrates involved in aldose–ketose transformation and hydroxyl groups in benzyl position were exposed to exchange reaction. Compared with ^1^H/D exchange, ^16^O/^18^O–exchange reactions proceed more slowly; however, the use of oxygen exchange for small molecules is associated with the formation of unstable products. The samples often have to be heated in an environment enriched with a heavier isotope up to 95 °C [23,24], which can lead to adverse reactions, such as pericyclic reactions [25], oxidation reactions [26] and reactions of radicals and carbenes [27], with the analyte destruction. It is also known that under the action of cytochromes P450 on drugs, the structure includes mainly nonexchangeable hydroxyl. These shortcomings limit the applicability of ^16^O/^18^O exchange for in vitro drug metabolism studies. The key to solving this problem may be performing the in vitro metabolism studies in the presence of an excess of ^18^O_2_, which makes it possible to obtain isotope-labeled compounds that are stable over time.

The first experiments on the insertion of the isotope oxygen-18 using hepatic microsomes appeared in the second half of the 20th century [28,29,30]. One of the first was J. Parley’s work on the mechanism of the oxidation of d-amphetamine by rabbit hepatic oxygenase [31]. Using isotope labeling and GC-MS, the authors showed that the initial step of oxidation is the hydroxylation of a substrate at the α-carbon to the amino group and concluded that molecular oxygen served as the oxygen source. Since then more than 50 years have passed, and mass spectrometry has developed: it is possible to combine a mass spectrometer and a liquid chromatograph, tandem mass spectrometers have been created and high-resolution instruments have appeared (Q-TOF, Orbitrap, ICR), which made the combination of chromatography with mass spectrometry one of the most versatile, sensitive, efficient and high-performance methods for studying the qualitative and quantitative composition of samples.

One of the first works on oxygen-18 isotope labeling with LM and high-resolution mass spectrometry (Q-TOF) was provided by X. Yang and W. Chen [32]. The authors studied hepatic microsomal metabolism of mGluR5 antagonist MTEP ((3-[2-methyl-1,3-thiazol-4-yl)ethynyl] pyridine). To confirm the mechanism of formation of the aldehyde M3 metabolite, incubation studies with ^18^O_2_ and H_2_^18^O were carried out, and a novel metabolism pathway for xenobiotics with thiazole moiety was suggested.

Here, we propose an approach based on carrying out two parallel hepatic microsomal drug metabolism studies: with and without the insertion of ^18^O isotope labels. Using bupivacaine as an example, we prove that this approach allows a significant increase in the degree of confidence in interpretating metabolic data and detecting minor metabolites that are usually ignored by analysis using HPLC in combination with high-resolution mass spectrometry (HPLC-HRMS).

## 2. Results and Discussion

**Figure 1:**
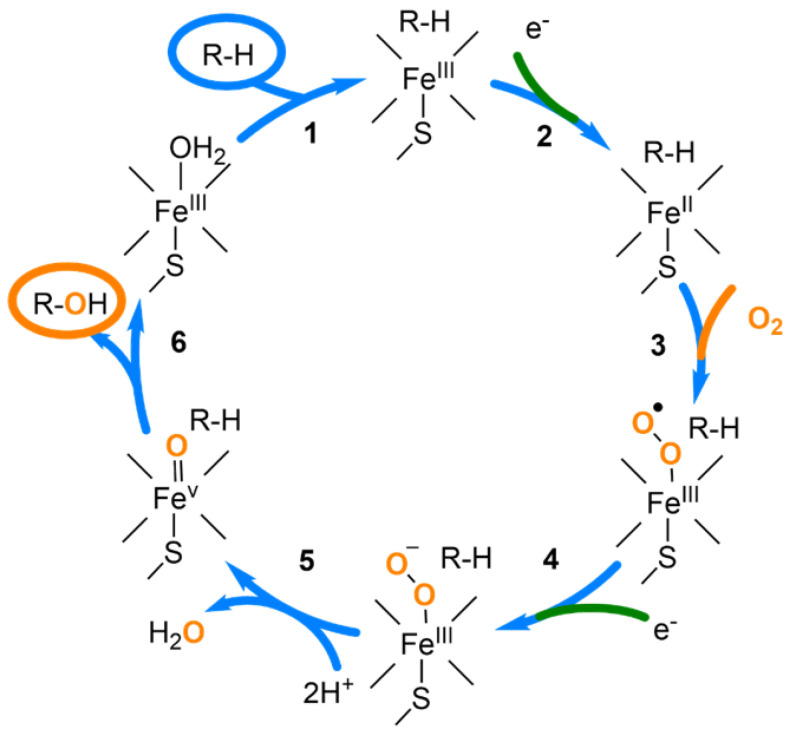
Generalized catalytic cycle for P450 reactions. R-H—parent drug (**1**); R-OH—hydroxy-metabolite (**6**).

The isotope labeling of metabolites with oxygen-18 is possible due to the oxidation mechanism of isoenzymes of cytochrome P450, contained in a suspension of rat liver microsomes (RLM) (Figure 1). This involves binding the dissolved oxygen to the ferrous enzyme [33], and if oxygen in Step 3 is ^18^O_2_, heavy isotope-labeled metabolites can be obtained in Step 6 (Figure 1).

When comparing the data of in vitro metabolism with RLM in air and in excess of ^18^O_2_, it is possible to clearly detect metabolites formed during drug oxidation by a characteristic delta of *Z* × 2.0043 *m/z*, where *Z* is the number of new oxygens after incubation with the in vitro system:

ΔM=Z×(M(O18)−M(O16))=Z×(17.9992−15.9949)=Z×2.0043 Da

We evaluated the capabilities of the ^18^O labeling approach on the example of bupivacaine. The drug’s metabolism was well investigated, and according to the European Medicines Agency assessment report for bupivacaine, 3-hydroxybupivacaine (3′-OH-bupivacaine) and 4-hydroxybupivacaine (4′-OH-bupivacaine) are its two major metabolites in rats [34]. Hedeland’s group [35] managed to detect 13 phase I metabolites, among which 11 (85%) could contain the oxygen-18 isotope label. The experiments were carried out on horses and *Cunninghamella* fungi, which might provide metabolic pathways different from rats, and the HPLC-MS was performed on a low-resolution instrument. Although the direct comparison of the results is restricted, this report was used as a reference for our work.

We managed to detect 34 metabolites with ^18^O-labeled groups: hydroxy- (H), dihydroxy- (DH), oxy- (O), hydroxy- with oxy- (HO), dealkylated with hydroxy- (DAH) and dealkylated with oxy- (DAO). The degree of isotopic labeling with oxygen-18 varied from 20% to 45%. (This range of values is probably due to the fact that the rates of formation of different types of metabolites vary, and the amount of dissolved heavy oxygen decreases throughout the experiment due to, for example, competition in solubility with the remaining oxygen-16.)

**Figure 2:**
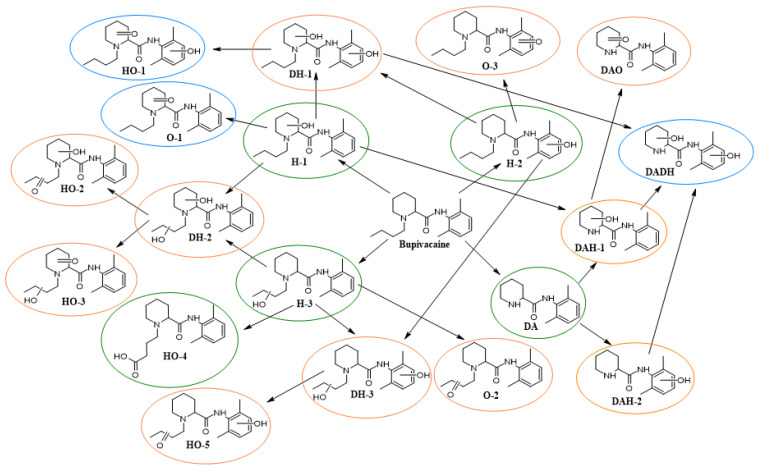
Bupivacaine metabolic pathways. The blue oval indicates undetected metabolites; the green oval indicates known metabolites found during the experiment; the orange oval indicates new previously unknown metabolites discovered during the experiment. Hydroxy- (H), dihydroxy- (DH), oxy- (O), hydroxy- with oxy- (HO), dealkylated with hydroxy- (DAH), dealkylated with oxy- (DAO).

**Figure 3:**
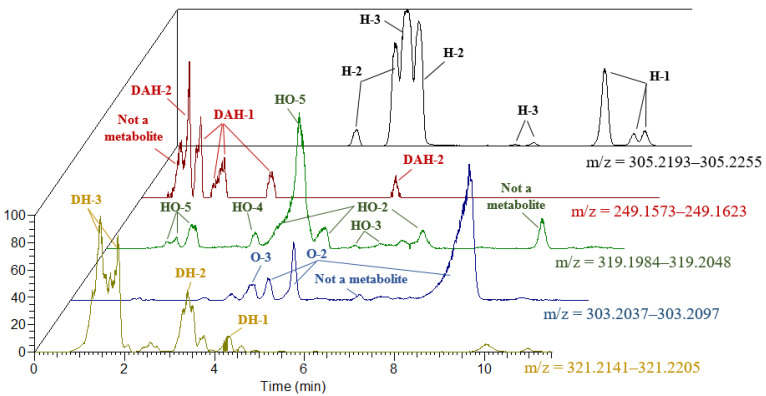
Extracted ion chromatograms of bupivacaine metabolites after incubation with RLM in the presence of excess ^18^O_2_ in positive-ion electrospray ionization mode. Hydroxy- (H), dihydroxy- (DH), oxy- (O), hydroxy- with oxy- (HO), dealkylated with hydroxy- (DAH), dealkylated with oxy- (DAO).

Metabolites’ structures and annotated mass chromatograms are shown in Figure 2 and Figure 3. The data of the analysis in positive ionization mode are presented in terms of the greater intensity of positive ions in comparison with their corresponding negative ions.

**Figure 4:**
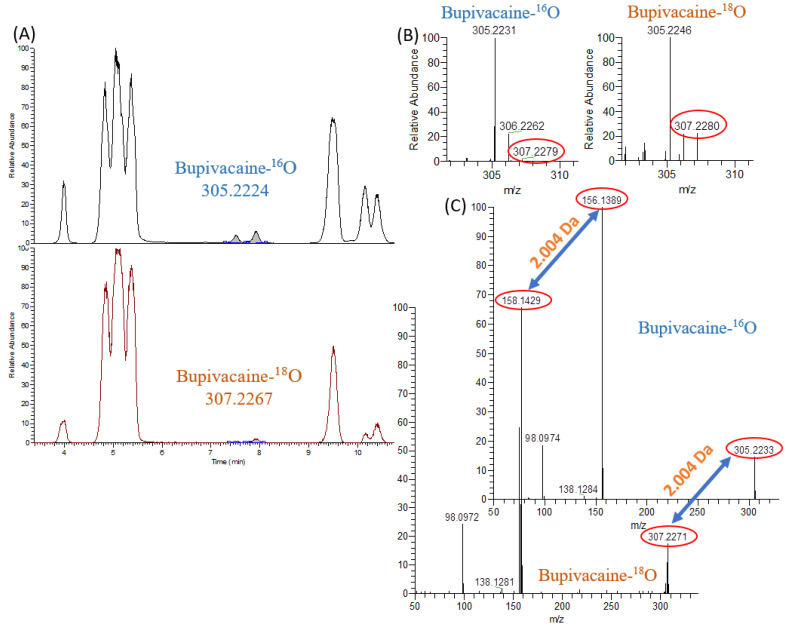
Comparison between incubation of bupivacaine in air and in excess of ^18^O_2_. (**A**) extracted ion chromatograms *m/z* 305.2224 (**top**) and 307.2267 (**bottom**). (**B**) MS spectra without isotope labeling (**left**) and oxygen-18 labeled (**right**). (**C**) MS/MS-spectra of H-3 metabolite with RT 7.51 min (similar to metabolites with RT 5.08 and 7.92 min) without isotope labeling (**top**) and oxygen-18 labeled (**bottom**), obtained in PRM mode after 20-fold concentration.

Using the ^18^O isotope label made it possible to reliably identify a significant number of compounds as metabolites, even for low-abundant signals without MS/MS-spectrum. For instance, the MS/MS spectra of signals with retention time (RT) 7.50 and 7.91 min in DDA mode was not obtained; however, the presence of the ^18^O label clearly indicated that the signals belong to the hydroxy-metabolites of bupivacaine (Figure 4A,B). To confirm the effectiveness of oxygen-18 isotope labeling for the detection of metabolites, the sample was concentrated 20 times, and the MS/MS spectrum in PRM mode was obtained. The signal with *m/z* = 156.1389 shifted by 2.004 Da to *m/z* = 158.1429 (Figure 4C).

In addition, this method made it possible to reliably determine previously undetected metabolites HO (Figure S1), DAH (Figure S2), DAO (Figure S3), O (Figure S4) and DH (Figure S5). The hypothetical structures of the obtained ions are shown in the spectra. For DAO, we observed a signal with *m/z* = 247.1447 shift to *m/z* = 249.1489 (Figure S3C). We also found that signal with *m/z* = 261.1605 does not correspond to a metabolite with a terminal COOH group (HO-4, Figure 2). As indicated in Heideland’s article (*m/z* = 261), it corresponds to C_15_H_21_N_2_O_2_ and occurs in MS/MS spectra of HO-5 (Figure S1D) and in others; however, the HO-4 metabolites were also formed (Figure S1C). Mass spectrometric data on metabolites are presented in Table 1, wherein HO-1 and dealkylated with dihydroxy- (DADH) metabolites were not found.

On the other hand, the use of oxygen-18 during the incubation of drugs with microsomes makes it possible to obtain stable labeled metabolites. During the study of the stability, it was found that H metabolites with ^18^O isotope labels can be stored for half a year. The area ratio of unlabeled hydroxy-metabolites/hydroxy-metabolites of bupivacaine with oxygen-18 isotope labels changed less than 10% over 6 months of storage at −20 °C (Table S1). The S_r_ total for freshly prepared 1 µM water solutions of bupivacaine chloride with a difference of 6 months was 6% (Table S2). 

**Figure 5:**
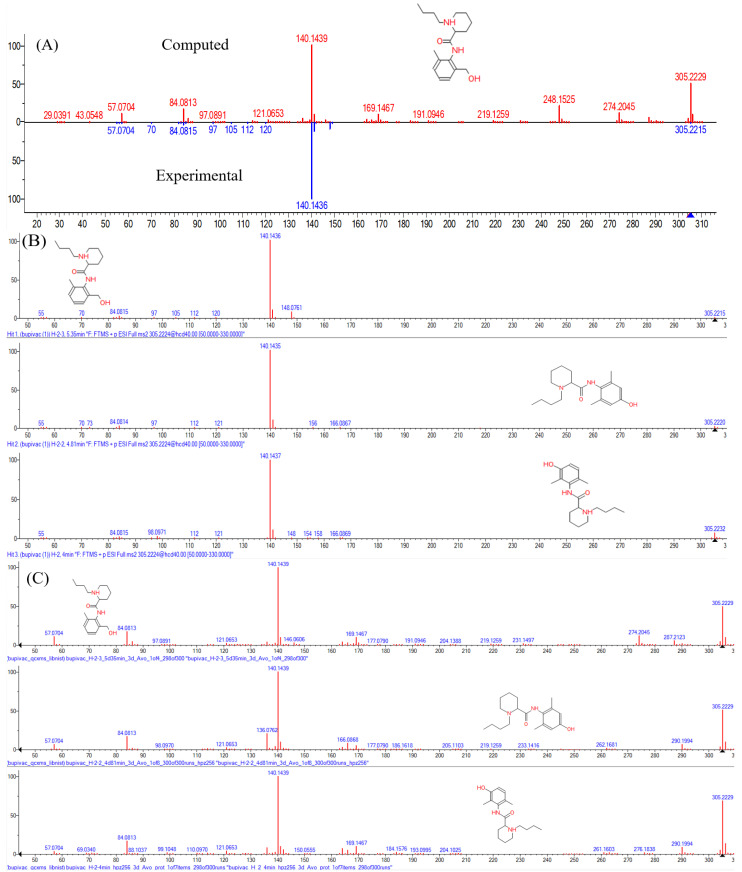
(**A**) A comparison between computed MS/MS spectrum and experimental for H-2 metabolite with RT 5.37 min. (**B**,**C**) Experimental and computed MS/MS spectrum for 5.37, 4.82 and 3.98 min (from top to bottom).

Despite a noticeable increase in the number of reliably detected metabolites, the method does not allow for determining the regioselectivity of the oxidative action of P-450 cytochromes. During the experiment, it was possible to identify classes of metabolites such as H-1, H-2 and H-3 for metabolites with the hydroxy group in cyclohexane, phenyl and butyl group, respectively, but not the position of the introduced hydroxyl. To solve this problem, we attempted to compare the obtained MS/MS spectra of hydroxy-metabolites (the major) with the quantum mechanical-computed spectra. We generated mass spectra for compounds with different positions of the hydroxyl group in cyclohexane (H-1), phenyl (H-2) and butyl group (H-3). Having selected the optimal parameters, we achieved a high degree of coincidence for the predicted and experimental spectra, for example, H-2 metabolite with RT 5.37 min (Figure 5A; read more in the Supplementary Materials). However, the experimental spectra of the same type metabolites (Figure 5B) as well as the calculated ones (Figure 5C) had only minor differences, which allowed us to conduct only group identification but did not allow us to distinguish individual metabolites inside the group.

Recently, several methods were proposed for retention time prediction in LC [36,37,38,39,40,41]. However, to use most of them, a training dataset of retention time values for at least hundreds of molecules is needed to adjust the models for new separation conditions. Since such a dataset was unavailable in the current work [42], we used the machine learning-based predictor described in our previous work to get retention time values of detected metabolites in different separation conditions (Table S3). On the assumption that the retention order is preserved in both conditions, we attempted to annotate the metabolites based on these predicted retention time values. However, we found some contradictions between the experimental and calculated retention times. For example, we observed that two H-2 metabolites with RT 3.98 and 4.82 min were eluted earlier than the metabolite of H-3 class with RT 5.08 min. We then observed that one H-2 metabolite with RT 5.37 min and, after that, two H-3 metabolites with RT 7.51 and 7.92 min were eluted. From 9.50 to 10.40 min, three H-1 metabolites were detected (Figure 3, Table 1). However, predicted RT values follow a different elution order. For oxy-metabolites, the O-3 metabolite was eluted earlier than any of the O-2 metabolites, whereas the predicted RT values indicate later elution of O-3. These and other contradictions did not allow using this approach to annotate bupivacaine metabolites.

Moreover, “tag dropout” can become a limiting factor. For example, it is known that hydroxyl groups at allyl positions can be exchanged with oxygen from H_2_O. It should be borne in mind, however, according to our earlier works [24], that the rate of such an exchange is not very high, and oxygen-18 isotope labels may be preserved.

## 3. Materials and Methods

### 3.1. Chemicals and Reagents

Analytical standard of bupivacaine hydrochloride, potassium hydrophosphate (K_2_HPO_4_), dihydrophosphate (KH_2_PO_4_), sodium chloride (NaCl), potassium chloride (KCl), magnesium chloride (MgCl_2_) and pooled liver microsomes from rat (Sprague-Dawley, male) were purchased from Merck KGaA (Darmstadt, Germany).

Gaseous ^18^O_2_ (92.0% ^18^O_2_–enriched) was purchased from ST Selivanenko O.I (Moscow, Russia).

HPLC solvents water (H_2_O), acetonitrile (ACN) and formic acid (HCOOH) were obtained from Merck KGaA (Darmstadt, Germany).

### 3.2. Experiment

**Figure 6:**
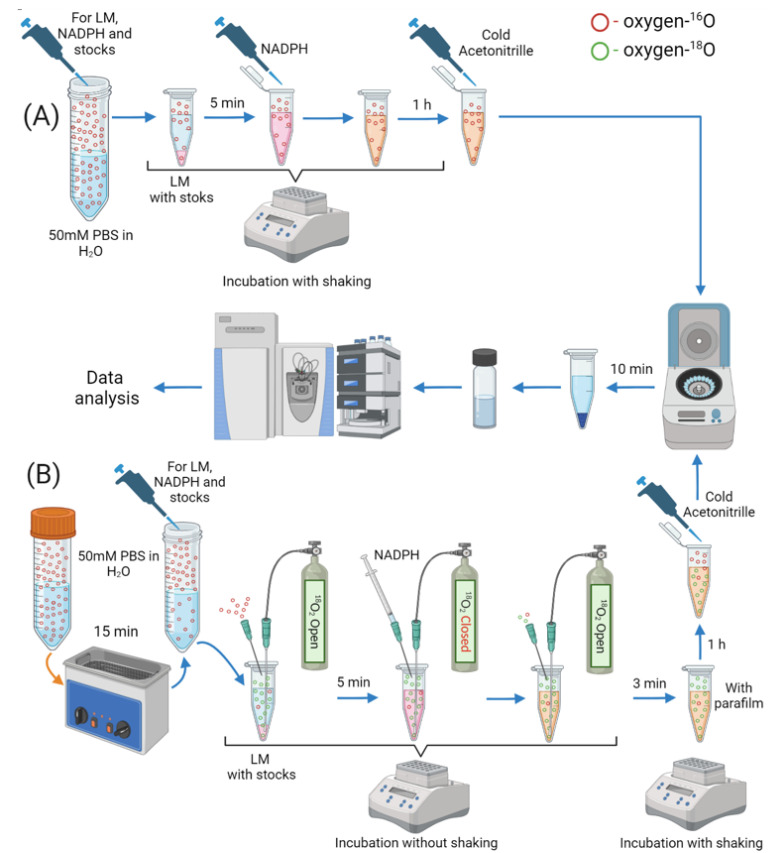
Stages of the microsomal stability experiment according to the standard protocol (**A**) and in the presence of ^18^O_2_ for labeling metabolites (**B**). LM—liver microsomes; PBS—phosphate buffered saline.

Bupivacaine was incubated in parallel under 2 conditions: A and B in Figure 6A and B, respectively. Experiments were carried out according to the standard protocol for in vitro drug metabolism stability with microsomes, described by Knigts et al. [5] and in more detail in this paper’s Supplementary Materials. In both cases, the incubation time was 1 h. To implement a better insertion of the isotope label in B (Figure 6B) before preparing solutions of microsomes, cofactors and reducing agent-NADPH, a 15 min degassing of the 50 mM phosphate buffered saline (PBS) in water was carried out. Degassing was performed at this stage, since the cytochromes P450 can be inactivated by sonication [43]. After that during the preincubation of hepatic microsomes with stocks, gaseous ^18^O_2_ was passed through the solution to displace ^16^O_2_. In order to compensate for excess pressure and carry out further injection of NADPH, a syringe needle was inserted into the lid of the vessel. Injection of NADPH followed by ^18^O_2_ passing through the solution was made. After 3 min, the needle was removed, and the hole was closed with parafilm to create an airtight vessel.

### 3.3. Sample Preparation

To prepare a working solution for HPLC-HRMS method development, 30 µL of incubated mixtures was diluted with 180 µL of acetonitrile followed by centrifugation for 10 min at 14,000× *g*. The supernatant fractions were poured into labeled HPLC tubes (or appropriate containers for the chosen analytical technique) for analysis.

### 3.4. HPLC-HRMS Conditions

All experiments were carried out with Dionex 3000 UltiMate coupled with QExactive Orbitrap (Bremen, Germany). We used Hypersil Gold C8 (2.1 × 50 mm, 1.8 µ) HPLC column. Mobile phase A involved 0.1% formic acid in 5% aqueous solution of acetonitrile. Mobile phase B involved 0.1% formic acid in acetonitrile delivered as the following gradient at a flow rate of 0.50 mL/min: 0–1.0 min 0% B, 1.0–10.5 min 0–10.0% B, 10.5–11.0 10–95% B, 11.0–12.0 min 95% B and 13.0–15.0 min 0% B. The injection volume was 1 μL. The resolving power was 35,000 (for *m/z* = 200). The Sheath, Aux and Spare gases were set to 45, 15 and 5, respectively. The Spray Voltage was 4.1 kV (for positive and negative ionization modes), the temperature of the desolvating capillary was 350 °C. S-Leans RF level was 50 and the source temperature was set to 200 °C.

Open screening for metabolites was performed by Full MS followed (100–900 *m/z*) by data-dependent analysis (DDA) both in positive and negative ionization modes, and targeted metabolite searches were carried out by parallel reaction monitoring (PRM). Control samples included an intact RLM sample, a RLM sample spiked with the substrate without cofactor, a RLM with cofactor without substrate and the substrate spiked to the PBS. To prevent HPLC column contamination between samples, blank solutions of acetonitrile were analyzed.

### 3.5. Oxygen-18 Label Stability Study

To determine the stability of the introduced isotope labels of oxygen-18 into the bupivacaine metabolites, samples and controls were incubated both in air and in excess of ^18^O_2_ and were reanalyzed after 6 months. Each sample was analyzed 3 times. The storage was carried out in HPLC vials at −20°C in a mixture 1:6 of 50 mM PBS (pH 7.4) in water and acetonitrile, respectively. The stability of the HPLC-HRMS system was controlled by analyzing a bupivacaine chloride standard solution. Freshly prepared 1 µM water solutions were analyzed 3 times before analysis of incubated samples.

### 3.6. Data Analysis

Spectra were processed using Xcalibur™ 4.4 software (Thermo Fisher Scientific, Houston, TX, USA). The search for metabolites was carried out both in automatic mode using the Compound Discoverer™ 3.2 program (Thermo Fisher Scientific, Houston, TX, USA) and manually. The interpretation of mass spectra was carried out manually as well as using the ScolmiX v2 open web application (https://skolmix.anvil.app/, accessed on 20 January 2023) developed by our scientific group. Received mass spectra were compared with quantum chemical-computed spectra for metabolite structures conducted by Conformer-Rotamer Ensemble Sampling Tool (CREST) [44] for protonation and QCxMS [45,46] for fragmentation of the molecules.

## 4. Conclusions

We have described the method and the protocol for the biocatalytic insertion of ^18^O-isotope labels in drug metabolites under the influence of microsomes in the presence of gaseous oxygen-18. More than 20 previously unreported metabolites of the local anesthetic bupivacaine have been reliably discovered and identified in the absence of their standards, and the operability of this approach for studying drug metabolism in vitro has been demonstrated using this method. In addition, this approach can be applied to the synthesis of isotopically labeled standards in cases where countersynthesis to obtain metabolites of complex natural bioorganic compounds is impossible or is much more laborious, time-consuming and costly. The use of this approach does not resolve the main limitations of mass spectrometry, such as reliable identification of optical and positional isomers of drug metabolites. At the same time, the possibility of the reverse isotope exchange reaction should be taken into consideration. However, the method allows for deepening drug metabolism study to reliably detect a larger number of metabolites, which biological activity will be evaluated in the subsequent stages of drug studies. 

### Associated Content

Preparation of stock solutions, microsomal suspension, cofactor mix and experiment stages are in the Supplementary Materials, as are annotated MS/MS spectra of HO (Figure S1), DAH (Figure S2), O (Figure S4), DH (Figure S5) and mass spectra of DAO (Figure S3). Data for assessing the stability of oxygen-18 isotope labels and the performance of the HPLC-HRMS system are in Tables S1 and S2, respectively. The conditions for predicting MS/MS spectra of metabolites by the quantum mechanical calculations are also presented in the Supplementary Materials.

## Figures and Tables

**Table 1:** A summary of metabolites found in the experiment with bupivacaine. The retention times of isomers that belong to the same type of metabolites are separated by semicolons.

Classes of Metabolites	RT of Detected Metabolites, min	[M+H]^+^, *m/z*	Characteristic Product Ions
H-1	9.50; 10.15; 10.40	305.2224	82.0659 (C_5_H_8_N); 96.0816 (C_6_H_10_N); 156.1393 (C_9_H_18_NO)
H-2	3.98; 4.82; 5.37		84.0815 (C_5_H_10_N); 98.0974 (C_6_H_12_N); 140.1440 (C_9_H_18_N)
H-3	5.08; 7.51; 7.92		84.0815 (C_5_H_10_N); 98.0974 (C_6_H_12_N); 156.1393 (C_9_H_18_NO)
DAH-1	1.30; 1.68; 1.83; 2.93	249.1598	82.0659 (C_5_H_8_N); 100.0764 (C_5_H_10_NO)
DAH-2	1.04; 6.38		84.0815 (C_5_H_10_N)
HO-1	not found	319.2016	not found
HO-2	4.25; 4.66; 7.00		82.0658 (C_5_H_8_N); 100.0764 (C_5_H_10_NO); 112.0763 (C_6_H_10_NO); 170.1180 (C;_9_H_16_NO_2_); 261.1604 (C_15_H_21_N_2_O_2_)
HO-3	5.10; 5.47		82.0658 (C_5_H_8_N); 170.1180 (C;_9_H_16_NO_2_); 259.1444 (C_15_H_19_N_2_O_2_)
HO-4	3.30		84.0815 (C_5_H_10_N); 96.0816 (C_6_H_10_N); 170.1180 (C;_9_H_16_NO_2_); 245.1653 (C_15_H_21_N_2_O)
HO-5	1.25; 1.40; 2.00; 4.35		84.0815 (C_5_H_10_N); 96.0816 (C_6_H_10_N); 154.1231 (C_9_H_16_NO); 261.1603 (C_15_H_21_N_2_O_2_)
O-1	not found	303.2067	not found
O-2	3.30; 5.00; 8.83		84.0815 (C_5_H_10_N); 96.0816 (C_6_H_10_N); 154.1232 (C_9_H_16_NO); 245.1653 (C_15_H_21_N_2_O)
O-3	3.00		84.0815 (C_5_H_10_N); 140.1440 (C_9_H_18_N) 259.1444 (C_15_H_19_N_2_O_2_)
DH-1	4.10	321.2173	96.0816 (C_6_H_10_N); 156.1393 (C_9_H_18_NO)
DH-2	3.29		84.0815 (C_5_H_10_N); 114.0920 (C_6_H_12_NO); 172.1337 (C_9_H_18_NO_2_)
DH-3	1.45; 1.87		84.0815 (C_5_H_10_N); 98.0974 (C_6_H_12_N); 156.1393 (C_9_H_18_NO)
DAO	5.61	247.1448	70.0658 (C4H8N); 98.0608 (C5H8NO); 122.0964 (C8H12N)
DADH	not found	not found	not found
DA	3.06	233.1648	84.0815 (C_5_H_10_N); 150.0920 (C_9_H_12_NO) detected without isotope labeling

## Data Availability

Not Applicable.

## Supplementary Materials

The following supporting information can be downloaded at https://www.mdpi.com/article/10.3390/ijms24054569/s1
