# Supplementary material for: Simple In Vitro 18O Labeling for Improved Mass Spectrometry-Based Drug Metabolites Identification: Deep Drug Metabolism Study
Source: Int J Mol Sci. 2023 Feb 26;24(5):4569. doi: 10.3390/ijms24054569 (PMC10002766; doi:10.3390/ijms24054569)
Supplement: Supplementary file 1 [file ijms-24-04569-s001.zip › ijms-2234511-supplementary.pdf]

Supplementary Materials to the manuscript

"Simple in vitro 18O labeling for improved mass spectrometry based drug metabolites identification: Deep drug metabolism study" by

*Boris Tupertsev[1,2], Sergey Osipenko[3], Albert Kireev[1], Eugene Nikolaev[1] and Yury Kostyukevich[1]**

[1]Center of Molecular and Cellular Biology (CMCB), Skolkovo Institute of Science and Technology, Nobel Str., 3, 121205 Moscow, Russia;

[2]Moscow Institute of Physics and Technology, Phystech School of Biological and Medical Physics, Institutskiy per., 9, Dolgoprudny, Moscow, 141701, Russia;

[3]Center for Computational and Data-Intensive Science and Engineering, Skolkovo Institute of Science and Technology, Nobel Str., 3, 121205 Moscow, Russia;

*Correspondence authors: y.kostyukevich@skoltech.ru

Preparation of stock solutions

Stock solutions was prepared in water for Bupivacaine hydrochloride at 10mM and diluted to 10 µM (1:1000 dilution) with 50mM PBS (pH 7.4) in water.

Stock solutions (10 mM) should be stored at −20°C or −80 °C for 3 months.

Preparation of microsomal suspension

Dilute 10 µL of LM suspension (20 mg/mL) with 156.7 µL PBS to final concentration of 1.2 mg/mL.

Preparation of cofactor mix

Dissolve 3.6 mg of NADPH in 216 µL of water to prepare 20 mM stock solution.

Dissolve 4.7 mg of $MgCl_2$ in 732 µL of water to prepare 66 mM stock solution.

Mix both solutions (1:1) to prepare 10x cofactor mix.

Experiment

Bupivacaine was incubated in parallel under two protocols:

A) To 40 µL of LM suspension in PBS (1.2 mg/mL) add 5 µL of 10 µM stock solution, vortex and incubate in shaker at 37°C and 600 rpm. After 5 min add 5 µL of co-factor mix, vortex and incubate 1 hour at 37°C and 600 rpm.

B) 50mM PBS (pH 7.4) in water degassed for 15 min. Solutions of stocks, microsomes and cofactors prepare similarly to the corresponding previous paragraphs. To 40µL of LM suspension in PBS (1.2 mg/mL) add 5µL of 10µM stock solution and incubate in shaker at 37°C pass gaseous oxygen-$^{18}O_2$ through the resulting solution. After 5 min add 5µL of co-factor mix and pass $^{18}O_2$

through the resulting solution for 3 min. Seal the container and incubate in shaker at 37°C and 600 rpm.

To prepare control sample add 5 μL of water instead of co-factor mix/microsomes.

Data Analysis

Compound Discoverer™ 3.2 program (Thermo Fisher Scientific, Houston, Texas, USA) workflow was MetID w Stats Expected and Unknown w Background Removal (Figure S6). As an expected compound bupivacaine structure was added. All I phase transformation were added for calculating. Mass tolerances of expected and detected compounds were set to 10ppm, RT tolerance 0.1 min. Scan filter setting DDA.

Computational details of ESI-MS/MS bupivacaine metabolites spectra:

Computations consist of two main steps: protonation and fragmentation. The computations were conducted on Ubuntu version 22.04 installed as a virtual machine on top of Windows 10 by VMware Workstation 16 Player with 16 computer cores of dual Intel Xeon E5-2680@v2 2.8 GHz and 124 GB RAM.

Protonation of the molecules was conducted by using the quantum chemistry based Conformer-Rotamer Ensemble Sampling Tool (CREST). The most populated protonated structures were selected by multilevel optimization with automated script CREST version 2.12 using the GFN2-xTB program compatible with xTB version 6.4.0. After crude and loose optimization remaining structures of protomers stand inside user defined 20 kcal/mol energy ranges.

Fragmentation was conducted by QCxMS version V5.2.0, which is a quantum chemical (QC) based program that enables users to calculate mass spectra (MS) using Born-Oppenheimer Molecular Dynamics (MD). It is the successor of the QCEIMS program, in which the EI part is exchanged to x (x=EI, CID) to account for the greater general applicability of the program.[1]. QCxMS runs in three main steps: a) the input structure is equilibrated at 500 K on a 5 ps molecular dynamics trajectory (MD) with a timestep of 0.5 fs, gas pressure was 1 mTorr as default value, b) 300 structural snapshots were taken along MD trajectory for sampling of the conformational space, c) conducting massively parallel calculations with the snapshot structures as starting geometries (production runs). In the production runs collisions between $[M+H]^+$ and neutral gas Ar atoms with a randomized collision angle are simulated for each product ion run (Comparison of computed spectra revealed better stability

---

[1] (1) Koopman, J.; Grimme, S. From QCEIMS to QCxMS: A Tool to Routinely Calculate CID Mass Spectra Using Molecular Dynamics. *J. Am. Soc. Mass Spectrom.* **2021**, *32* (7), 1735–1751. https://doi.org/10.1021/jasms.1c00098.

of computation process and better match with experimental spectra when Ar was used rather than N_2). If the critical energy E0 is reached, statistical and non-statistical fragmentation of the molecular ion occurs. To induce sufficient dissociation of $[M+H]^+$, the collision process had to be repeated multiple times, number of collisions determined automatically by preinstalled script of QCxMS.

The ESI-MS/MS spectra of the most populated protomers were calculated by using the general activation run-type with a collision cell length of 0.25 m, collision energy of 60 eV, full-auto run-type specifics, and 300 number of trajectories.

Initial structures of bupivacaine metabolites were optimized with molecular mechanics force field MMFF94s (number of steps 5000, steepest descent algorithm, convergence $10e^{-7}$) by using Avogadro free software, version 1.2.0 with embedded Open Babel version 2.3.90. [https://avogadro.cc/.]

Table S1. The results of the analysis of bupivacaine chloride with rat liver microsomes samples incubated in air and in excess of oxygen-18 at the first day of the experiment and after 6 months storage at −20°C. C.u , are area conventional units obtained by Q Exactive Orbitrap mass spectrometer

Classes of metabolites	^{16}O 07.02.2022		^{18}O 07.02.2022		$^{16}O/^{18}O$ 1d	^{16}O 08.08.2022		^{18}O 08.08.2022		$^{16}O/^{18}O$ 6m	Δ, %
	RT, min	Area, c.u.	RT, min	Area, c.u.		RT, min	Area, c.u.	RT, min	Area, c.u.		
H-2	3.99	30297774	4.00	1169838	26	4,07	22442796	4,08	935870	25	8
	4.84	118878162	4.84	8696010	14	4,93	89382077	4,92	7012911	13	7
H-3	5.05	181444887	5.09	15152132	12	5,13	138507547	5,18	12219461	11	6
H-2	5.37	124406748	5.37	9805619	13	5,47	95697498	5,46	8103817	12	7
H-3	7.51	4820959	7.51	83253	58	7,59	3571081	7,58	66602	54	8
	7.91	7682714	7.91	207130	37	7,99	5733369	7,98	167040	34	8
H-1	9.48	134098484	9.51	5341168	25	9,56	98601826	9,57	4272934	25	9
	10.14	42610907	10.15	356390	120	10,23	32777621	10,25	294537	111	7
	10.40	37340246	10.39	939393	40	10,48	27865855	10,48	746735	37	7

Table S2. The results of the analysis of two 1µM freshly prepared water solutions of bupivacaine chloride at the first day of the experiment and after 6 months. C.u , are area conventional units obtained by Q Exactive Orbitrap mass spectrometer

Sample	RT, min	Area, c.u.	S_r, %
1.1 Bupivacaine chloride 1µM	11.32	566075744	6
1.2 Bupivacaine chloride 1µM	11.33	569114585	
1.3 Bupivacaine chloride 1µM	11.32	560735491	
2.1 Bupivacaine chloride 1µM*	11.40	503482917	
2.2 Bupivacaine chloride 1µM*	11.40	512105360	
2.3 Bupivacaine chloride 1µM*	11.39	507348574	

*- freshly prepared solution after 6 months

Table S3. Retention time prediction for bupivacaine metabolites using Skolmix RT predictor

SMILES metabolite structure	Predicted RT, min
**HO-2**	
Cc1cccc(C)c1NC(=O)C1C(O)CCCN1CCCC=O	11,38
Cc1cccc(C)c1NC(=O)C1CC(O)CCN1CCCC=O	11,23
Cc1cccc(C)c1NC(=O)C1CCC(O)CN1CCCC=O	11,54
Cc1cccc(C)c1NC(=O)C1CCCC(O)N1CCCC=O	11,75
O=C(Nc1c(C)cccc1C)C1CCCC[N+]1(O)CCCC=O	13,18

Cc1cccc(C)c1NC(=O)C1C(O)CCCN1CCC(C)=O	10,21
Cc1cccc(C)c1NC(=O)C1CC(O)CCN1CCC(C)=O	9,82
Cc1cccc(C)c1NC(=O)C1CCC(O)CN1CCC(C)=O	10,63
Cc1cccc(C)c1NC(=O)C1CCCC(O)N1CCC(C)=O	10,66
O=C(Nc1c(C)cccc1C)C1CCCC[N+]1(O)CCC(C)=O	12,80
O=C(CN1CCCC(O)C1C(=O)Nc1c(C)cccc1C)CC	11,10
O=C(CN1CCC(O)CC1C(=O)Nc1c(C)cccc1C)CC	10,64
O=C(CN1CC(O)CCC1C(=O)Nc1c(C)cccc1C)CC	11,09
O=C(CN1C(CCCC1O)C(=O)Nc1c(C)cccc1C)CC	11,42
O=C(Nc1c(C)cccc1C)C1CCCC[N+]1(O)CC(=O)CC	12,97
OC1CCCN(C(=O)CCC)C1C(=O)Nc1c(C)cccc1C	12,49
OC1CC(C(=O)Nc2c(C)cccc2C)N(CC1)C(=O)CCC	11,27
OC1CCC(C(=O)Nc2c(C)cccc2C)N(C1)C(=O)CCC	12,52
OC1CCCC(C(=O)Nc2c(C)cccc2C)N1C(=O)CCC	12,10
O=C(Nc1c(C)cccc1C)C1CCCC[N+]1(O)C(=O)CCC	11,55
**HO-3**	
O=C1CCCN(C(O)CCC)C1C(=O)Nc1c(C)cccc1C	13,26
O=C1CC(C(=O)Nc2c(C)cccc2C)N(CC1)C(O)CCC	11,83
O=C1CCC(C(=O)Nc2c(C)cccc2C)N(C1)C(O)CCC	12,96
OC(CCC)N1C(=O)CCCC1C(=O)Nc1c(C)cccc1C	11,83
OC(CN1CCCC(=O)C1C(=O)Nc1c(C)cccc1C)CC	11,04
OC(CN1CCC(=O)CC1C(=O)Nc1c(C)cccc1C)CC	11,12
OC(CN1CC(=O)CCC1C(=O)Nc1c(C)cccc1C)CC	10,95
OC(CN1C(=O)CCCC1C(=O)Nc1c(C)cccc1C)CC	10,46
Cc1cccc(C)c1NC(=O)C1C(=O)CCCN1CCC(C)O	11,94
Cc1cccc(C)c1NC(=O)C1CC(=O)CCN1CCC(C)O	10,99
Cc1cccc(C)c1NC(=O)C1CCC(=O)CN1CCC(C)O	10,87
Cc1cccc(C)c1NC(=O)C1CCCC(=O)N1CCC(C)O	11,53
Cc1cccc(C)c1NC(=O)C1C(=O)CCCN1CCCCO	10,28
Cc1cccc(C)c1NC(=O)C1CC(=O)CCN1CCCCO	9,08
Cc1cccc(C)c1NC(=O)C1CCC(=O)CN1CCCCO	9,65
Cc1cccc(C)c1NC(=O)C1CCCC(=O)N1CCCCO	8,96
O=C(Nc1c(C)cccc1C)C1C(=O)CCC[N+]1(O)CCCC	14,49
O=C(Nc1c(C)cccc1C)C1CC(=O)CC[N+]1(O)CCCC	14,46
O=C(Nc1c(C)cccc1C)C1CCC(=O)C[N+]1(O)CCCC	16,06
O=C1CCCC(C(=O)Nc2c(C)cccc2C)[N+]1(O)CCCC	13,87
**HO-4**	
Cc1cccc(C)c1NC(=O)C1CCCCN1CCCC(=O)O	9,76
**HO-5**	
O=C(CCC)N1CCCCC1C(=O)Nc1c(C)cccc1CO	11,89
O=C(CCC)N1CCCCC1C(=O)Nc1c(C)ccc(O)c1C	13,43
O=C(CCC)N1CCCCC1C(=O)Nc1c(C)cc(O)cc1C	12,18
O=C(CN1CCCCC1C(=O)Nc1c(C)cccc1CO)CC	10,57

O=C(CN1CCCCC1C(=O)Nc1c(C)ccc(O)c1C)CC	11,91
O=C(CN1CCCCC1C(=O)Nc1c(C)cc(O)cc1C)CC	10,41
Cc1cccc(CO)c1NC(=O)C1CCCCN1CCC(C)=O	9,38
Cc1ccc(O)c(C)c1NC(=O)C1CCCCN1CCC(C)=O	12,09
Cc1cc(O)cc(C)c1NC(=O)C1CCCCN1CCC(C)=O	10,59
Cc1cccc(CO)c1NC(=O)C1CCCCN1CCCC=O	10,69
Cc1ccc(O)c(C)c1NC(=O)C1CCCCN1CCCC=O	13,31
Cc1cc(O)cc(C)c1NC(=O)C1CCCCN1CCCC=O	11,82
**H-1**	
Cc1cccc(C)c1NC(=O)C1CCCC(O)N1CCCC	13,26
Cc1cccc(C)c1NC(=O)C1CCC(O)CN1CCCC	13,57
Cc1cccc(C)c1NC(=O)C1CC(O)CCN1CCCC	8,51
Cc1cccc(C)c1NC(=O)C1C(O)CCCN1CCCC	12,37
O=C(Nc1c(C)cccc1C)C1CCCC[N+]1(O)CCCC	15,19
**H-2**	
Cc1cccc(CO)c1NC(=O)C1CCCCN1CCCC	12,08
Cc1ccc(O)c(C)c1NC(=O)C1CCCCN1CCCC	14,72
Cc1cc(O)cc(C)c1NC(=O)C1CCCCN1CCCC	13,01
**H-3**	
OC(CCC)N1CCCCC1C(=O)Nc1c(C)cccc1C	12,37
OC(CN1CCCCC1C(=O)Nc1c(C)cccc1C)CC	10,93
Cc1cccc(C)c1NC(=O)C1CCCCN1CCC(C)O	11,11
Cc1cccc(C)c1NC(=O)C1CCCCN1CCCCO	9,54
**O-2**	
Cc1cccc(C)c1NC(=O)C1CCCCN1CCCC=O	11,67
Cc1cccc(C)c1NC(=O)C1CCCCN1CCC(C)=O	10,34
O=C(CN1CCCCC1C(=O)Nc1c(C)cccc1C)CC	11,53
O=C(CCC)N1CCCCC1C(=O)Nc1c(C)cccc1C	12,73
**O-3**	
Cc1cccc(C=O)c1NC(=O)C1CCCCN1CCCC	13,54
**DAH-1**	
O=C(Nc1c(C)cccc1C)C1CCCC(O)N1	10,15
O=C(Nc1c(C)cccc1C)C1CCC(O)CN1	10,61
O=C(Nc1c(C)cccc1C)C1CC(O)CCN1	9,64
O=C(Nc1c(C)cccc1C)C1NCCCC1O	9,60
O=C(Nc1c(C)cccc1C)C1CCCCN1O	12,28
**DAH-2**	
O=C(Nc1c(C)cccc1CO)C1CCCCN1	10,51
O=C(Nc1c(C)ccc(O)c1C)C1CCCCN1	11,78
O=C(Nc1c(C)cc(O)cc1C)C1CCCCN1	10,59
**DH-1**	
Cc1cccc(CO)c1NC(=O)C1C(O)CCCN1CCCC	12,13
Cc1ccc(O)c(C)c1NC(=O)C1C(O)CCCN1CCCC	13,09

Cc1cc(O)cc(C)c1NC(=O)C1C(O)CCCN1CCCC	11,62
Cc1cccc(CO)c1NC(=O)C1CC(O)CCN1CCCC	9,63
Cc1ccc(O)c(C)c1NC(=O)C1CC(O)CCN1CCCC	10,54
Cc1cc(O)cc(C)c1NC(=O)C1CC(O)CCN1CCCC	9,09
Cc1cccc(CO)c1NC(=O)C1CCC(O)CN1CCCC	13,17
Cc1ccc(O)c(C)c1NC(=O)C1CCC(O)CN1CCCC	14,23
Cc1cc(O)cc(C)c1NC(=O)C1CCC(O)CN1CCCC	12,37
Cc1cccc(CO)c1NC(=O)C1CCCC(O)N1CCCC	12,86
Cc1ccc(O)c(C)c1NC(=O)C1CCCC(O)N1CCCC	14,64
Cc1cc(O)cc(C)c1NC(=O)C1CCCC(O)N1CCCC	13,17
O=C(Nc1c(C)cccc1CO)C1CCCC[N+]1(O)CCCC	14,95
O=C(Nc1c(C)ccc(O)c1C)C1CCCC[N+]1(O)CCCC	16,35
O=C(Nc1c(C)cc(O)cc1C)C1CCCC[N+]1(O)CCCC	15,13
**DH-2**	
O=C(Nc1c(C)cccc1C)C1C(O)CCC[N+]1(O)CCCC	14,18
OC1CCCN(C(O)CCC)C1C(=O)Nc1c(C)cccc1C	12,18
OC(CN1CCCC(O)C1C(=O)Nc1c(C)cccc1C)CC	10,61
Cc1cccc(C)c1NC(=O)C1C(O)CCCN1CCC(C)O	11,59
Cc1cccc(C)c1NC(=O)C1C(O)CCCN1CCCCO	10,44
O=C(Nc1c(C)cccc1C)C1CC(O)CC[N+]1(O)CCCC	14,22
OC1CC(C(=O)Nc2c(C)cccc2C)N(CC1)C(O)CCC	11,55
OC(CN1CCC(O)CC1C(=O)Nc1c(C)cccc1C)CC	11,79
Cc1cccc(C)c1NC(=O)C1CC(O)CCN1CCC(C)O	11,62
Cc1cccc(C)c1NC(=O)C1CC(O)CCN1CCCCO	9,52
O=C(Nc1c(C)cccc1C)C1CCC(O)C[N+]1(O)CCCC	14,92
OC1CCC(C(=O)Nc2c(C)cccc2C)N(C1)C(O)CCC	12,05
OC(CN1CC(O)CCC1C(=O)Nc1c(C)cccc1C)CC	10,93
Cc1cccc(C)c1NC(=O)C1CCC(O)CN1CCC(C)O	11,74
Cc1cccc(C)c1NC(=O)C1CCC(O)CN1CCCCO	9,82
O=C(Nc1c(C)cccc1C)C1CCCC(O)[N+]1(O)CCCC	14,90
OC1CCCC(C(=O)Nc2c(C)cccc2C)N1C(O)CCC	11,61
OC(CN1C(CCCC1O)C(=O)Nc1c(C)cccc1C)CC	10,72
Cc1cccc(C)c1NC(=O)C1CCCC(O)N1CCC(C)O	9,92
Cc1cccc(C)c1NC(=O)C1CCCC(O)N1CCCCO	10,18
O=C(Nc1c(C)cccc1C)C1CCCC[N+]1(O)C(O)CCC	12,03
O=C(Nc1c(C)cccc1C)C1CCCC[N+]1(O)CC(O)CC	13,01
O=C(Nc1c(C)cccc1C)C1CCCC[N+]1(O)CCC(C)O	13,03
O=C(Nc1c(C)cccc1C)C1CCCC[N+]1(O)CCCCO	11,56
**DH-3**	
O=C(Nc1c(C)cccc1CO)C1CCCC[N+]1(O)CCCC	14,95
OC(CCC)N1CCCCC1C(=O)Nc1c(C)cccc1CO	11,94
OC(CN1CCCCC1C(=O)Nc1c(C)cccc1CO)CC	11,01
Cc1cccc(CO)c1NC(=O)C1C(O)CCCN1CCC(C)O	11,18

Cc1cccc(CO)c1NC(=O)C1C(O)CCCN1CCCCO	10,87
O=C(Nc1c(C)ccc(O)c1C)C1CCCC[N+]1(O)CCCC	16,35
OC(CCC)N1CCCCC1C(=O)Nc1c(C)ccc(O)c1C	13,33
OC(CN1CCCCC1C(=O)Nc1c(C)ccc(O)c1C)CC	12,00
Cc1ccc(O)c(C)c1NC(=O)C1C(O)CCCN1CCC(C)O	12,24
Cc1ccc(O)c(C)c1NC(=O)C1C(O)CCCN1CCCCO	11,09
O=C(Nc1c(C)cc(O)cc1C)C1CCCC[N+]1(O)CCCC	15,13
OC(CCC)N1CCCCC1C(=O)Nc1c(C)cc(O)cc1C	12,09
OC(CN1CCCCC1C(=O)Nc1c(C)cc(O)cc1C)CC	10,46
Cc1cc(O)cc(C)c1NC(=O)C1C(O)CCCN1CCC(C)O	10,89
Cc1cc(O)cc(C)c1NC(=O)C1C(O)CCCN1CCCCO	9,84
**DAO**	
O=C1CCCC(N1)C(=O)Nc1c(C)cccc1C	11,49
O=C(Nc1c(C)cccc1C)C1CCC(=O)CN1	12,35
O=C(Nc1c(C)cccc1C)C1CC(=O)CCN1	10,60
O=C(Nc1c(C)cccc1C)C1NCCCC1=O	9,96

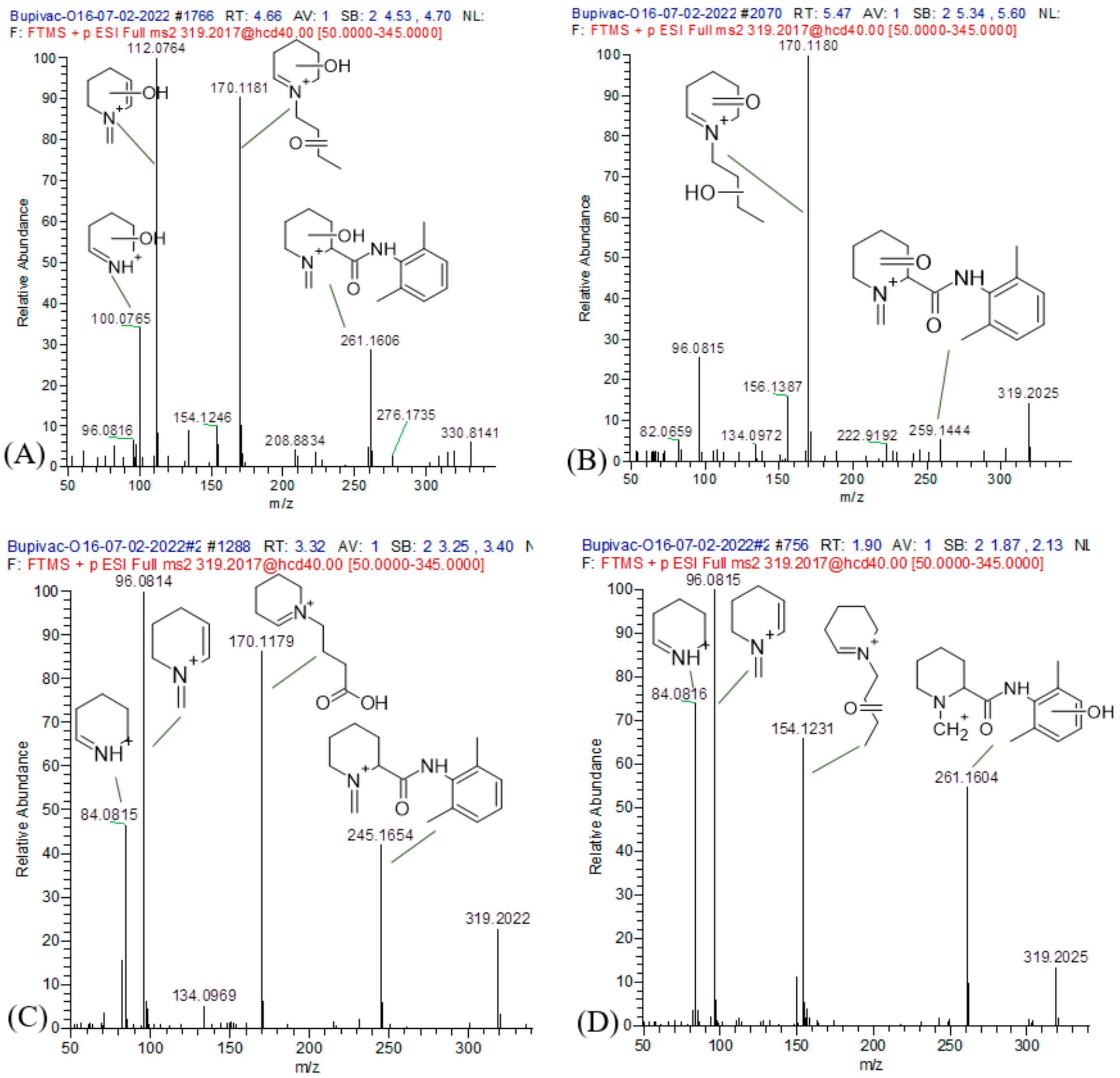

Figure S1. MS/MS spectra of unreported metabolites (**A**) HO-2 with RT 4.66 min, (**B**) HO-3 with RT 5.47, (**C**) known HO-4 with terminal carboxylic group RT 3.30 min and (**D**) HO-5 with RT 1.90 min. The presented spectra without oxygen-18 isotope labels in positive-ion electrospray ionization mode

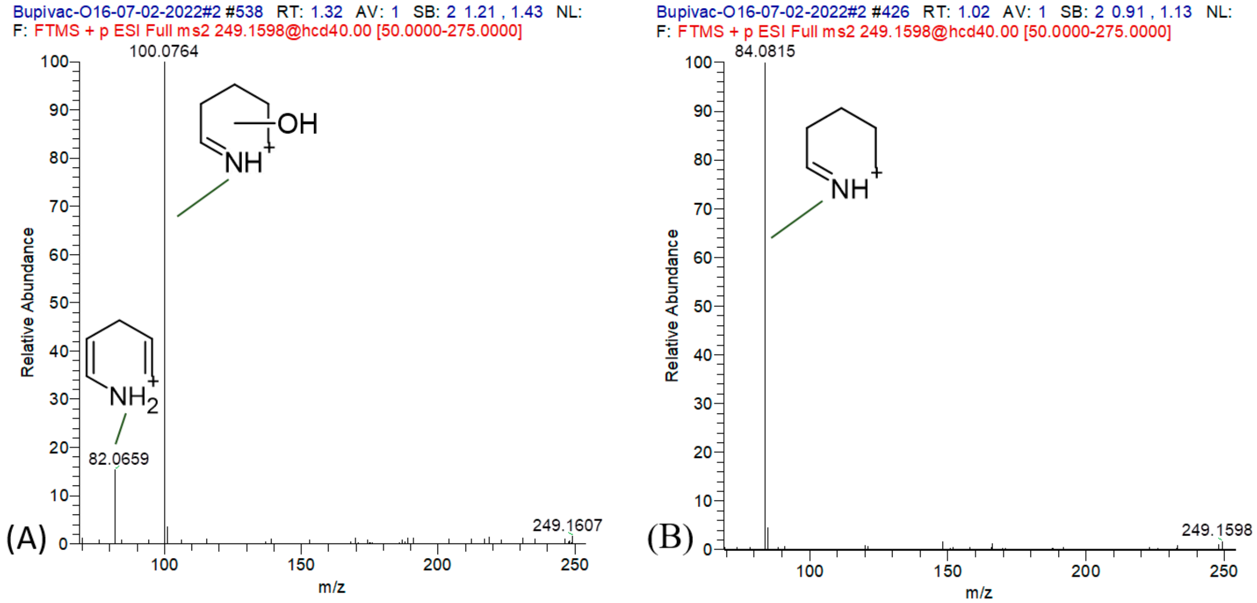

Figure S2. MS/MS spectra of unreported metabolites (**A**) DAH-1 with RT 1.32 min, (**B**) DAH-2 with RT 1.02. The presented spectra without oxygen-18 isotope labels in positive-ion electrospray ionization mode

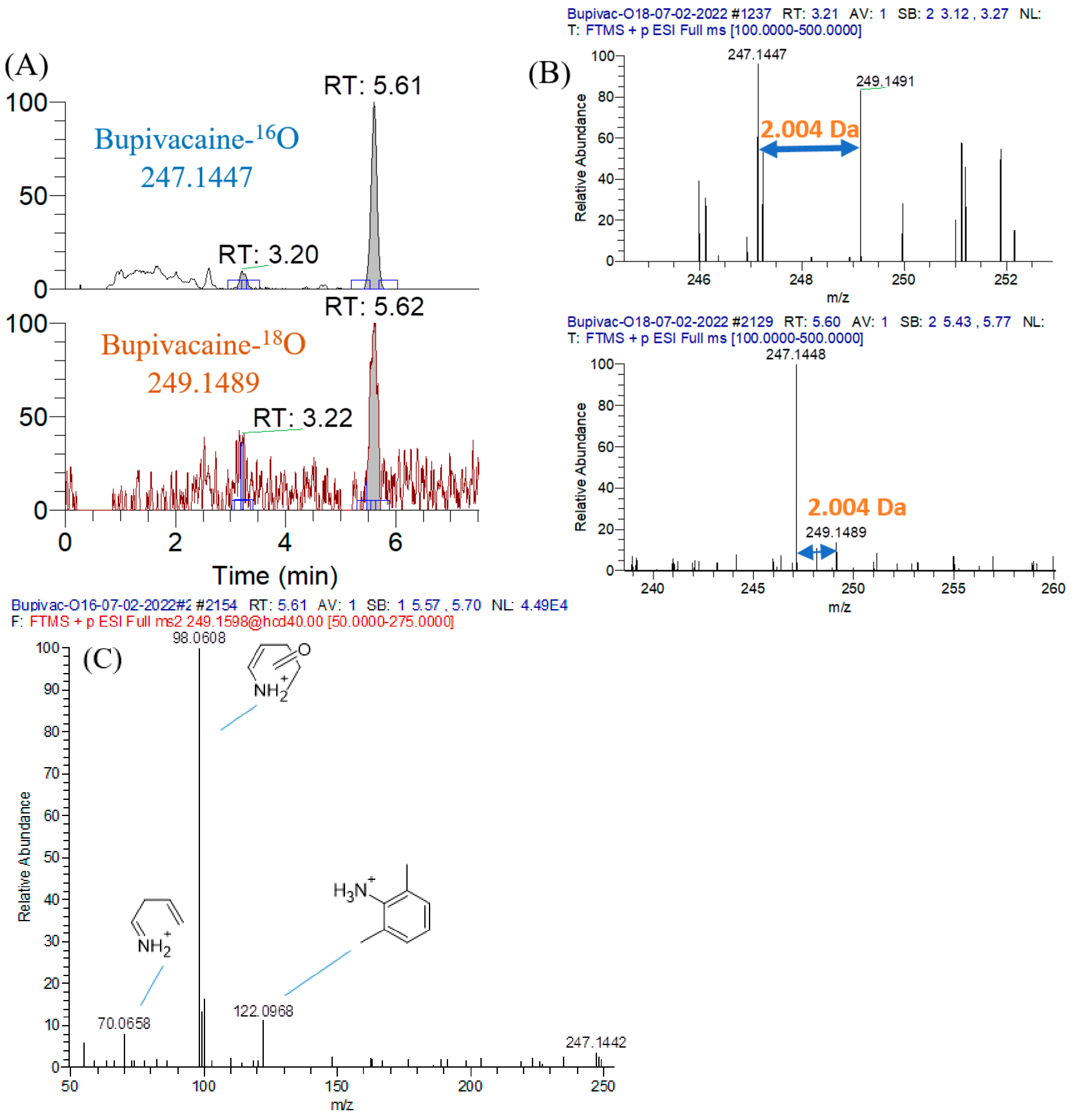

Figure S3. (**A**) Extracted ion chromatograms m/z 247.1441 and 249.1484 (for ^{18}O-isotope labeled),(**B**) mass spectra of unreported bupivacaine metabolites DAO with RT 3.20 and 5.61 min in positive-ion electrospray ionization mode. (**C**) MS/MS spectra DAO with RT 5.61 min

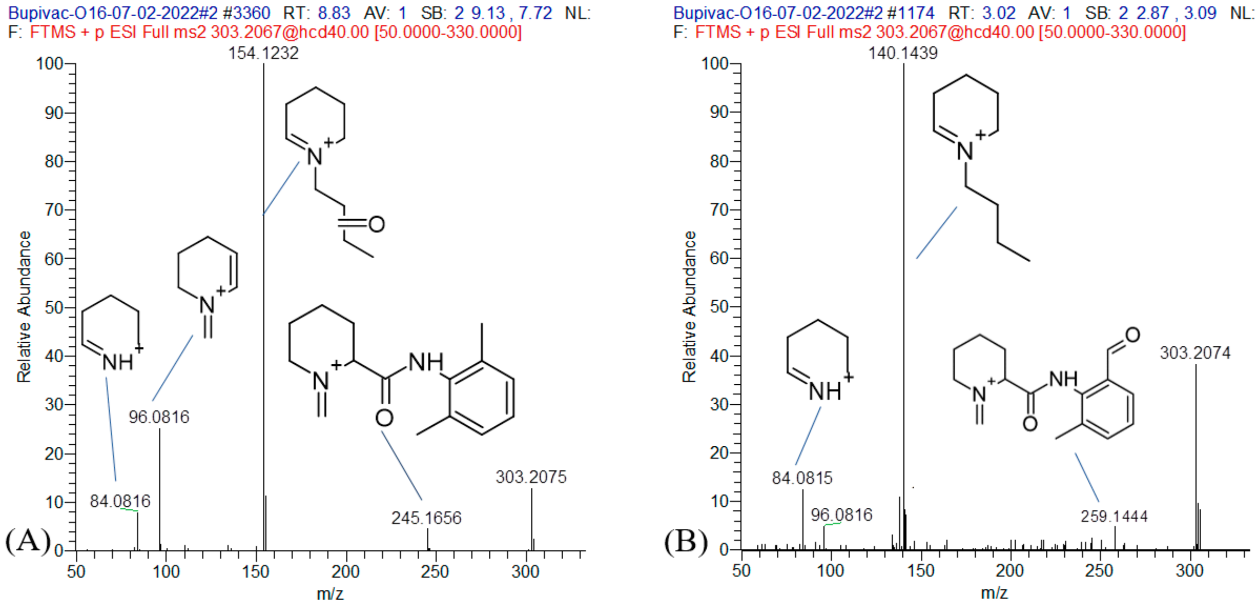

Figure S4. MS/MS spectra unreported metabolites (**A**) O-2 with RT 8.83 min and (**B**) O-3 with RT 3.00 min. The presented spectra without oxygen-18 isotope labels in positive-ion electrospray ionization mode

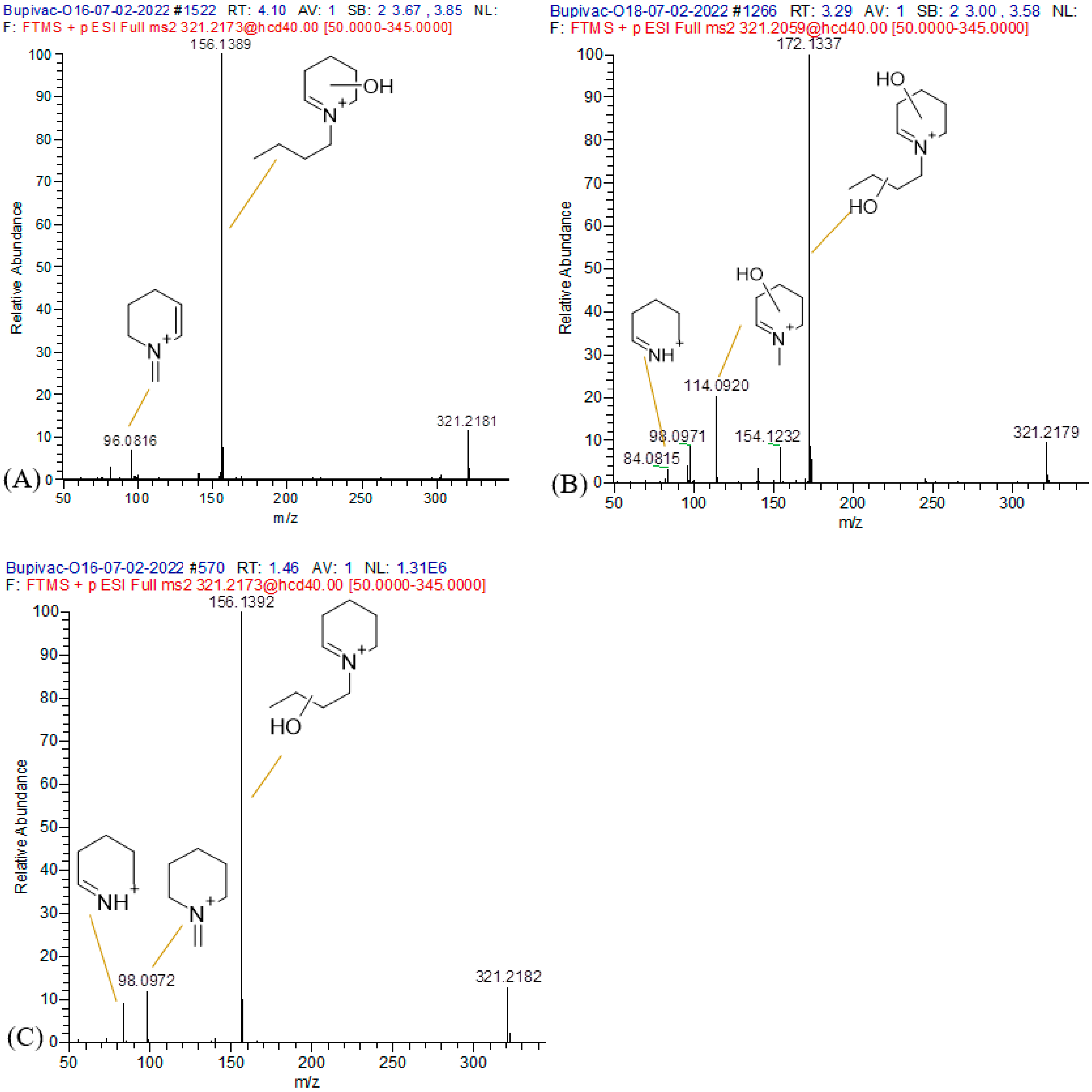

Figure S5. MS/MS spectra unreported metabolites (**A**) DH-1 with RT 4.10 min, (**B**) DH-2 with RT 3.29 min and (**C**) DH-3 with RT 1.45 min. The presented spectra without oxygen-18 isotope labels in positive-ion electrospray ionization mode

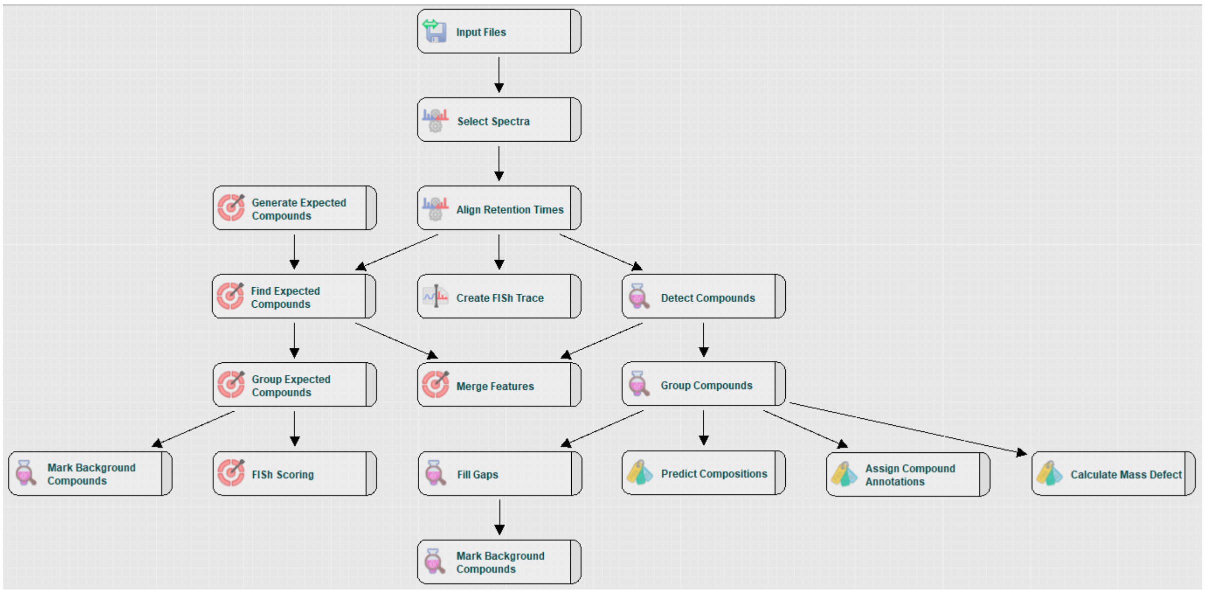

Figure S6. Compound Discoverer™ 3.2 (Thermo Fisher Scientific, Houston, Texas, USA) program workflow for bupivacaine metabolism study
